# Alterations in oxidative, inflammatory and apoptotic events in short-lived and long-lived mice testes

**DOI:** 10.18632/aging.100875

**Published:** 2016-01-23

**Authors:** María Eugenia Matzkin, Johanna Gabriela Miquet, Yimin Fang, Cristal Monique Hill, Daniel Turyn, Ricardo Saúl Calandra, Andrzej Bartke, Mónica Beatriz Frungieri

**Affiliations:** ^1^ Instituto de Biología y Medicina Experimental, CONICET, Vuelta de Obligado 2 Ciudad de Buenos Aires, Argentina; ^2^ Cátedra de Bioquímica Humana, Facultad de Medicina, Universidad de Buenos Aires, Ciudad de Buenos Aires, Argentina; ^3^ Departamento de Química Biológica, Instituto de Química y Fisicoquímica Biológicas, Facultad de Farmacia y Bioquímica, Universidad de Buenos Aires, Ciudad de Buenos Aires, Argentina; ^4^ Geriatrics Research, Department of Internal Medicine, School of Medicine, Southern Illinois University, Springfield, IL 62794, USA; ^5^ Department of Medical Microbiology, Immunology and Cell Biology, School of Medicine, Southern Illinois University, Springfield, IL 62794, USA

**Keywords:** aging, testis, inflammation, oxidative stress, apoptosis

## Abstract

Aged testes undergo profound histological and morphological alterations leading to a reduced functionality. Here, we investigated whether variations in longevity affect the development of local inflammatory processes, the oxidative state and the occurrence of apoptotic events in the testis. To this aim, well-established mouse models with delayed (growth hormone releasing hormone-knockout and Ames dwarf mice) or accelerated (growth hormone-transgenic mice) aging were used.

We hereby show that the testes of short-lived mice show a significant increase in cyclooxygenase 2 expression, PGD2 production, lipid peroxidation, antioxidant enzymes expression, local macrophages and TUNEL-positive germ cells numbers, and the levels of both pro-caspase-3 and cleaved caspase-3. In contrast, although the expression of antioxidant enzymes remained unchanged in testes of long-lived mice, the remainder of the parameters assessed showed a significant reduction.

This study provides novel evidence that longevity confers anti-inflammatory, anti-oxidant and anti-apoptotic capacities to the adult testis. Oppositely, short-lived mice suffer testicular inflammatory, oxidative and apoptotic processes.

## INTRODUCTION

Aging constitutes a universal, multi-factorial, progressive and irreversible process. There are conditioning intrinsic and extrinsic factors affecting the overall aging process. Among the former, both genetic and endocrine factors play key roles in aging. Over the years, growth hormone (GH) signaling has emerged as one of the well-established pathways involved in the regulation of aging and lifespan. Results obtained in natural mutant, gene knockout or transgenic mice demonstrate a remarkable negative association of somatotropic signaling and lifespan. Hence, mice with over-expression of GH have a reduced life expectancy [1]. In addition, increased levels of pro-inflammatory cytokines, enhanced expression of markers of renal and hepatic inflammation, altered ovarian, colonic and cardiac apoptosis, as well as decreased activity of the antioxidant enzyme catalase in the liver and kidney were reported in animals over-expressing GH [2-7].

On the contrary, mice with mutations that cause GH deficiency or GH resistance live longer than their genetically normal siblings (ranging from 25% to over 60%). These long-lived mice include animals with targeted disruption of the GH receptor gene [5], mice with defective anterior pituitary development (Ames dwarf mice) [8] and mice with a mutation of the GH releasing hormone gene [9]. GH-related mutant mice not only display extended longevity but they also show delayed onset of puberty, reduced chronic low-grade inflammatory activity, increased resistance to oxidative stress in endothelial cells, liver, kidney and heart, as well as decreased expression of apoptosis-related genes in skeletal muscle [10-14].

Regarding reproductive aging in males, literature on testicular function deficit during aging has been, thus far, limited and controversial. However, it has been established that aged testes undergo profound histological and morphological alterations [15,16] which ultimately lead to decreased steroidogenesis and, concomitantly, reduced spermatogenesis [17,18]. However, male reproductive functions do not cease abruptly during aging. In fact, the decreased androgen production and spermatogenesis during aging, does not result in infertility as there is evidence that advanced aged men have been able to father children [19].

Some reports indicate that aged rat Leydig cells express higher levels of cyclooxygenase 2 (COX2), a key inducible isoenzyme in prostaglandin (PG) synthesis. It is well-known that PGs belong to a group of pro-inflammatory molecules [4,20]. Moreover, COX2/PGs have been proposed to play a role in the age-related decline of testosterone production [21].

Relevant to male (in)fertility, we have reported that although COX2 is not detected in human testes with no evident morphological changes or abnormalities, it is expressed in testicular biopsies of men with impaired spermatogenesis and male infertility [22]. In fact, recent studies indicated that testicular inflammation and a pro-oxidant environment are common underlying factors in idiopathic infertility. This is reflected mainly by the expression of COX2 and an increase in the number of testicular immune cells (e.g: (MAC), mast cells) in biopsies from men with impaired spermatogenesis compared to biopsies with no abnormal spermatogenesis [22-24]. According to our data, the main somatic cell populations of the testes, i.e. Leydig and Sertoli cells, express COX2. Nevertheless, we have also seen that testicular MACs express COX2, as well as pro-inflammatory cytokines (IL1β and TNFα), anti-oxidant enzymes (superoxide dismutase 1, catalase and peroxiredoxin 1) and pro-/anti-apoptotic proteins (Bax and Bcl-2) [23,24]. In addition, there seems to be a close, yet not totally understood, cross-talk between COX2-derived products and reactive oxygen species (ROS). In this context, a recent study reported that 15d-PGJ2 induces the generation of ROS in the human testis [25]. Data collected from several groups reveal that an altered redox status by over-production of ROS might also account for the decreased LH receptor–adenylyl cyclase signaling circuit that characterizes aged Leydig cells [26-28].

On the other hand, ROS and the resulting oxidative stress might play a pivotal role in apoptosis. In this context, increased apoptotic events have been described in the human testis during aging [15].

In view of all the features of the GH-related mutants and GH-transgenic mice that we have highlighted earlier, mice with altered aging become useful and available experimental models that allow for the assessment of tissular consequences of changes in longevity. Thus, in this study, we used mice with delayed (GHRH-KO and Ames dwarf mice) or accelerated (GH-transgenic mice) aging to examine whether variations in longevity affect the development of local inflammatory processes, the oxidative state and the occurrence of apoptotic events in the testis. To this aim, COX2 expression, PGD2 production, lipid peroxidation, antioxidant enzymes expression, pro-apoptotic caspase-3 levels and a quantification of local MACs and TUNEL-positive germ cells have been assessed in these mouse models testes.

## RESULTS

### Longevity is inversely associated with testicular COX2 expression and PGD2 production

**Figure 1:**
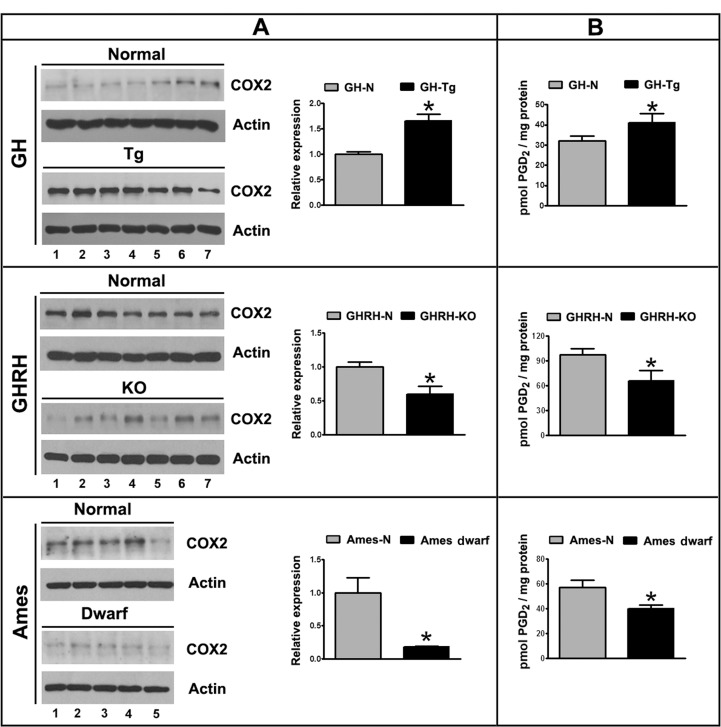
Longevity is inversely associated with testicular COX2 expression and PGD2 production Panel (**A**) COX2 (72 kDa) expression was evaluated by immunoblotting in testicular homogenates of short-lived (GH-Tg) and long-lived (GHRH-KO and Ames dwarf) mice. Bar plot graphs represent the mean ± S.E.M. and depict the quantification by densitometry of the bands. Results were normalized to actin (42 kDa) and expressed as fold change relative to the control (normal littermates), which was assigned a value of 1. Bar plot graphs represent the mean + SEM; n = 5-7. * p < 0.05, t-Student test. Panel (**B**) PGD2 testicular levels were determined by immunoassay in testicular homogenates from short-lived (GH-Tg) and long-lived (GHRH-KO and Ames dwarf) mice. Bar plot graphs represent the mean + SEM; n = 5-7. * p < 0.05, t-Student test.

COX2 expression was evaluated in testicular homogenates by immunoblotting. Results clearly indicate that COX2 protein levels are significantly higher in testes of short-lived (GH-Tg) mice than in control animals (Figure 1A). In contrast, GHRH-KO and Ames dwarf mice with extended longevity showed significantly decreased testicular COX2 expression in comparison to control littermates (Figure 1A). The analysis of testicular PGD2 levels revealed that production of this eicosanoid is enhanced in short-lived mice (GH-Tg) compared to their normal littermates (Figure 1B). In addition, lower levels of PGD2 were detected in testes from GHRH-KO and Ames dwarf long-lived mice (Figure 1B).

### COX2 is expressed in testicular macrophages and Leydig cells

In order to examine which cell populations of the testis might be contributing to COX2 expression, immunohistochemistry analyses were performed in testicular sections of short-lived (GH-Tg) and long-lived (GHRH-KO and Ames dwarf) mice.

**Figure 2:**
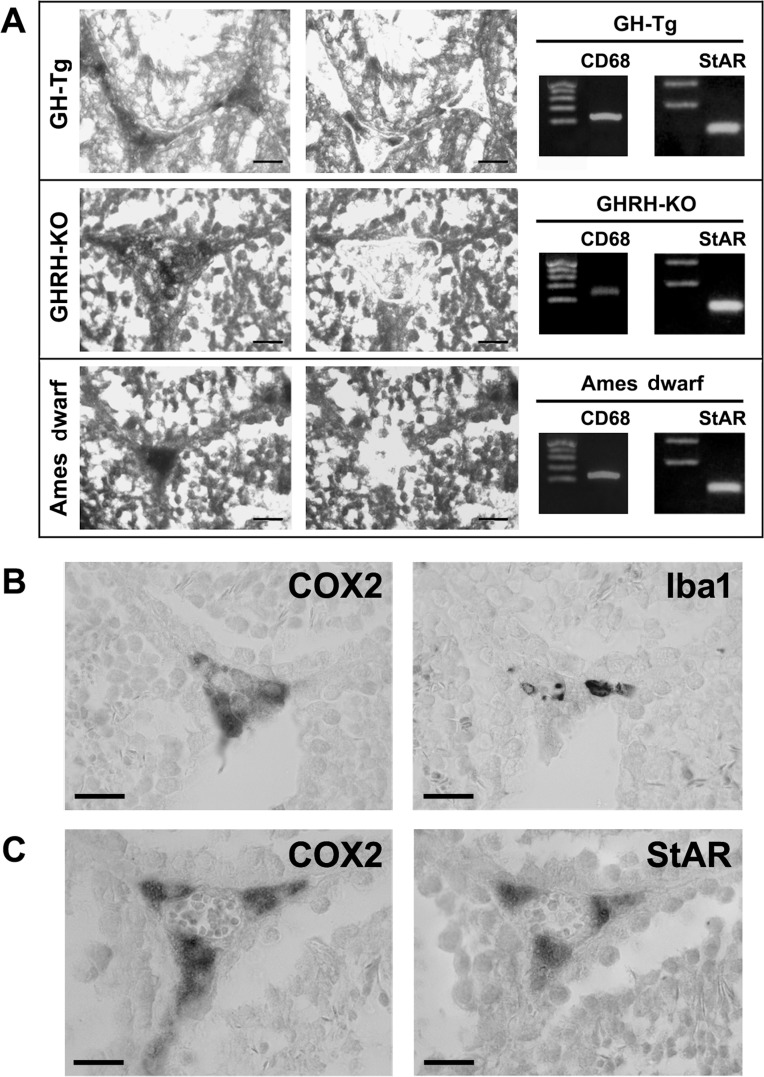
COX2 is expressed in testicular macrophages and Leydig cells Panel (**A**) COX2-immunoreactive interstitial cells were isolated by Laser Capture Microdissection (LCM) from testicular sections of short-lived (GH-Tg) and long-lived (Ames dwarf and GHRH-KO) mice. The same section is illustrated before (left panel) and after (right panel) LCM. Bar, 25 μm. A total of 50 to 80 COX2-immunopositive interstitial cells were isolated by LCM and subsequently used to evaluate the expression of CD68 (macrophage cell marker) and StAR (Leydig cell marker) by RT-PCR. Panels (**B** and **C**) Immuno-colocalization of COX2 and Iba1 (macrophage cell marker; Panel (**B**) and immuno-colocalization of COX2 and StAR (Leydig cell marker; Panel (**C**) in testicular sections from a short-lived mouse (GH-Tg) was examined using a light microscope. Bar, 20 (m. Similar images were seen when testicular sections from long-lived (Ames dwarf and GHRH-KO) mice were used (data not shown).

COX2-immunoreactive cells were primarily localized in the interstitium. Laser capture microdissection LCM followed by reverse transcription (RT)-PCR analyses and sequencing, revealed that the COX2-immunoreactive interstitial cell population is comprised of MACs and Leydig cells, as determined by the expression of the specific cell markers CD68 and StAR which recognize MACs and Leydig cells, respectively (Figure 2A).

Furthermore, immunohistochemical analyses performed on consecutive testicular sections revealed that some, but not all, COX2-immunoreactive interstitial cells were also positively stained for the MAC cell marker Iba1 Figure 2B). Positive immunostaining for COX2 was also detected in StAR-immunoreactive Leydig cells (Figure 2C). When COX2, Iba1 or StAR antiserum were omitted, immunostaining was not detected (data not shown).

### Longevity is inversely associated with testicular overall macrophage cell number and expression of macrophage markers

**Figure 3:**
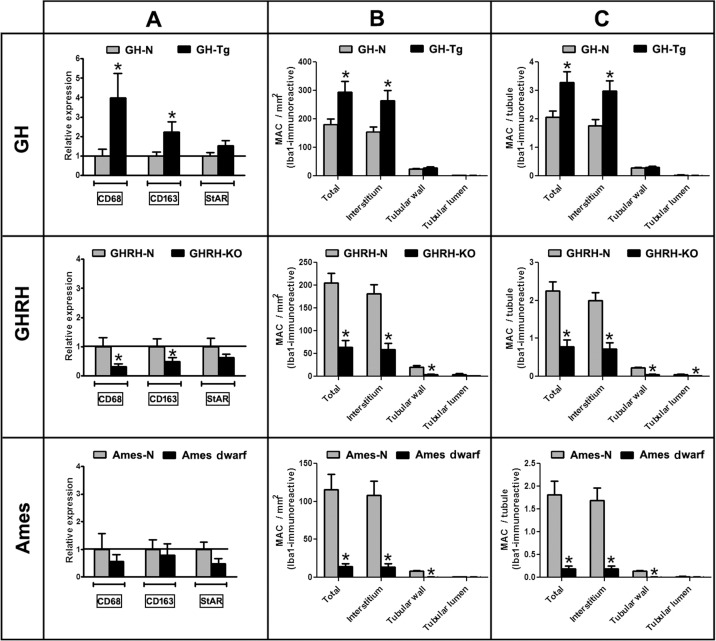
Longevity is inversely associated with testicular overall macrophage cell number and expression of macrophage markers Panel (**A**) the relative expression levels of CD68 and CD163 (macrophage cell markers) and the relative expression levels of StAR (Leydig cell marker) were determined by real time-PCR in testicular homogenates of short-lived (GH-Tg) and long-lived (GHRH-KO and Ames dwarf) mice and their normal littermates. Results were normalized to GAPDH housekeeping gene and expressed as fold change relative to the control (normal littermates), which was assigned a value of 1. Bar plot graphs represent the mean + SEM; n = 5-7. * p < 0.05, t-Student test. Panels (**B** and **C**) Quantification of Iba1-positive macrophages in testes of GH-Tg short-lived mice, GHRH-KO and Ames dwarf long-lived mice and their normal littermates was evaluated using a light microscope with a magnification of 400x and a gridded eyepiece. Results are expressed as macrophages/mm^2^ (Panel **B**) and macrophages/tubule (Panel **C**). Bar plot graphs represent the mean + SEM; n = 5-7. * p < 0.05, t-Student test.

Expression of cell-marker genes for macrophages and Leydig cells was evaluated by real time-PCR. In short-lived (GH-Tg) mice testes, there was a 4-fold increase in the expression of CD68, an infiltrating MACs cell marker, and a 2.2-fold increase in the expression of CD163, a resident MACs cell marker. In contrast, CD68 and CD163 expression was reduced by half in GHRH-KO long-lived mice testes compared to normal animals (Figure 3A). There was a trend toward lower CD68 and CD163 mRNA expression levels in the testis of Ames dwarf long-lived mice than in control littermates, although no actual statistical differences were observed (Figure 3A). No statistically significant differences were observed in the expression of the Leydig cell marker StAR in any of the experimental groups (Figure 3A).

Total numbers of Iba1-immunoreactive testicular MACs/mm^2^ were increased by 161% in mice with reduced longevity (GH-Tg). Interstitial MACs solely accounted for this increase (Figure 3B).

In Ames dwarf and GHRH-KO short-lived mice, total testicular MACs were critically reduced by 88% and 69%, respectively (Figure 3B). Interestingly, MACs located not only in the interstitium but also in the tubular wall were less abundant in GHRH-KO and Ames dwarf short-lived mice compared to their normal siblings (Figure 3B).

Same results were obtained when data were expressed as MACs/tubule (Figure 3C). Moreover, the number of MACs/tubule located in the tubular lumen was also decreased in GHRH-KO short-lived mice (Figure 3C).

### Decreased longevity is associated with increased testicular lipid peroxidation and antioxidant enzymes expression

**Figure 4:**
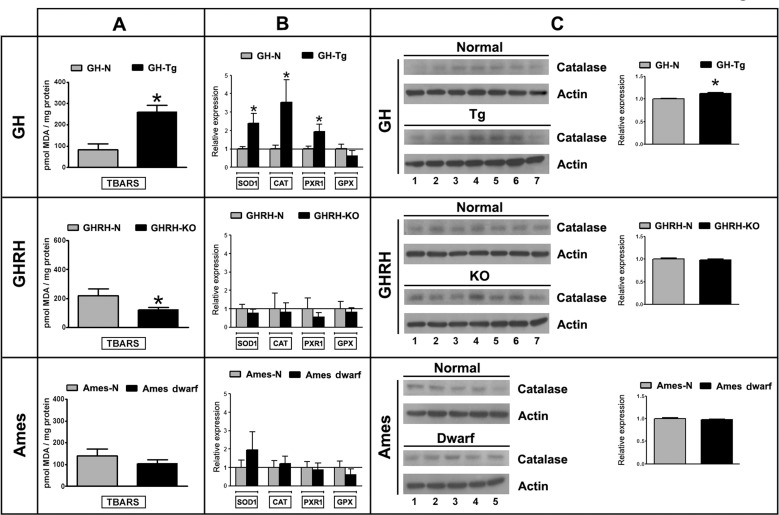
Decreased longevity is associated with increased testicular lipid peroxidation and antioxidant enzymes expression Panel (**A**) testicular lipid peroxidation was determined using the Thiobarbituric Acid Reactive Substances (TBARS) assay. Bar plot graphs represent the mean + SEM; n = 5-7. * p < 0.05, t-Student test. Panel (**B**) testicular mRNA expression of superoxide dismutase 1 (SOD1), catalase (CAT), peroxiredoxin1 (PXR1) and glutathione peroxidase (GPX) was determined by real time-PCR. Results were normalized to GAPDH housekeeping gene and expressed as fold change relative to the control (normal littermates), which was assigned a value of 1. Bar plot graphs represent the mean + SEM; n = 5-7. * p < 0.05, t-Student test. Panel (**C**) Testicular protein expression of catalase (60 kDa) was measured by immunoblotting. Bar plot graphs represent the mean ± S.E.M. and depict the quantification by densitometry of the bands. Results were normalized to actin (42 kDa) and expressed as fold change relative to the control (normal littermates), which was assigned a value of 1 (n = 5-7). * p < 0.05, t-Student test.

There was a 3.15-fold increase in Thiobarbituric Acid Reactive Substances TBARS production, a non-specific marker of lipid peroxidation and oxidative stress, in GH-Tg short-lived mice testes compared to their normal siblings (Figure 4A). Lower levels of TBARS were observed in GHRH-KO and Ames dwarf long-lived mice testes, although no actual statistically significant differences were seen in the latter group compared to their normal siblings (Figure 4A).

Antioxidant enzymes expression was evaluated by real time-PCR (Figure 4B) and immunoblotting (Figure 4C). GH-Tg short-lived mice testes displayed higher mRNA levels of superoxide dismutase 1 (SOD1), peroxiredoxin 1 (PXR1) and catalase (CAT), as well as higher CAT protein expression than their normal counterparts (Figures 4B, 4C). Glutathione peroxidase (GPX) mRNA levels remained unchanged in GH-Tg short-lived mice testes (Figure 4B).

No statistically significant differences were detected in the expression of antioxidant enzymes in GHRH-KO and Ames dwarf long-lived mice as compared to the corresponding normal controls (Figures 4B, 4C).

### Longevity is inversely associated with testicular apoptosis

**Figure 5:**
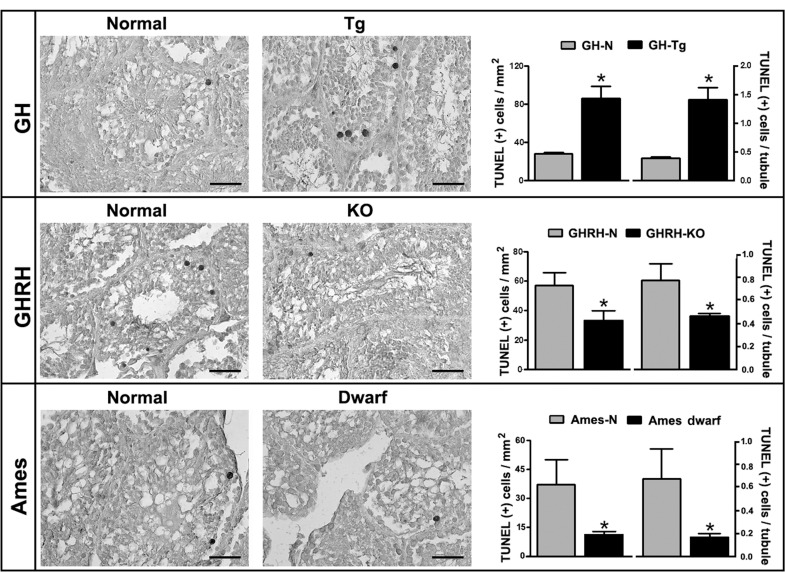
Longevity is inversely associated with testicular apoptosis Quantification of apoptotic cells in testes of GH-Tg short-lived mice, GHRH-KO and Ames dwarf long-lived mice and their normal littermates was determined by TUNEL assay using a light microscope with a magnification of 400x and a gridded eyepiece. Results are expressed as TUNEL-positive cells/mm^2^ and TUNEL-positive cells/tubule. Bar plot graphs represent the mean + SEM; n = 5-7. * p < 0.05, t-Student test.

Testes from GH-Tg mice with reduced longevity showed a 3-fold increase in TUNEL-positive cells (Figure 5). Apoptotic cells were exclusively located within the seminiferous tubule and showed typical features of germ cells. On the other hand, apoptosis was significantly reduced in GHRH-KO and Ames dwarf long-lived mice testes compared to their normal counterparts. Overall mice with increased longevity displayed only a few apoptotic cells (Figure 5).

**Figure 6:**
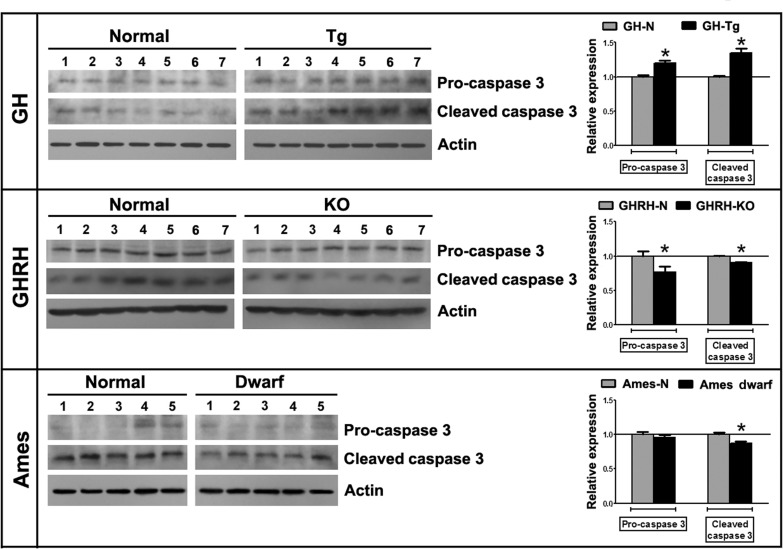
Longevity is inversely associated with testicular apoptosis Testicular expression of pro-caspase 3 (32 kDa) and cleaved caspase 3 (15 kDa) was measured by immunoblotting in testicular homogenates of short-lived (GH-Tg) and long-lived (GHRH-KO and Ames dwarf) mice and their normal littermates. Bar plot graphs represent the mean ± S.E.M. and depict the quantification by densitometry of the bands. Results were normalized to actin (42 kDa) and expressed as fold change relative to the control (normal littermates), which was assigned a value of 1. Bar plot graphs represent the mean + SEM; n = 5-7. * p < 0.05, t-Student test.

Caspase-3 protein levels were measured by immunoblotting in testes from GH-Tg short-lived mice, GHRH-KO and Ames dwarf long-lived mice and their normal littermates using an antibody that recognizes, both, pro-caspase-3 and cleaved caspase-3 forms. Our studies showed higher levels of both activated caspase-3 and pro-caspase-3 in testes from GH-Tg short-lived mice (Figure 6).

Caspase 3 and pro-caspase-3 expression levels were decreased in testes from GHRH-KO long-lived mice (Figure 6). Ames dwarf long-lived mice testes expressed lower levels of cleaved caspase-3, while pro-caspase-3 levels remained unchanged (Figure 6).

## DISCUSSION

This study provides novel evidence concerning the development of inflammatory status, oxidative stress and apoptotic events in testes of mice with extended/reduced longevity.

During aging, testicular function is dramatically altered. Early studies have demonstrated that testicular fragments, as well as Leydig cells purified from aged Brown Norway rats exhibit a reduced maximal hCG-stimulated testosterone production compared to those of young adults [27,36]. Numerous age-related changes, including the age-dependent decrease in steroidogenic capacity, have been associated to an increase in the inflammatory status of the tissue. It is well-known that COX2 expression is induced by cytokines and growth factors, particularly at sites of inflammation [37]. In this context, COX2 participation in the aged-Leydig cell phenotype has been suggested since these cells express higher levels of this inducible isoenzyme. Moreover, pharmacological inhibition of COX2 increases StAR expression and steroidogenesis [4,20].

To our knowledge, this is the first attempt to use well-established mouse models with delayed or accelerated aging to link testicular COX2 expression to reproductive longevity. In this study, we detected COX2 expression in long- and short-lived mouse testes mainly localized to Leydig cells and testicular MACs. COX2-immunoreactive Leydig cells have already been described by our group in testes of young adult hamsters [33,38] and patients suffering from idiopathic infertility [39]. In this study, using mouse models with altered lifespan, we hereby show an inverse association between longevity and testicular COX2 expression. Thus, our results further support the notion that age-related changes in eicosanoids production are associated with alterations of the inflammatory state. As stated previously, there is an increasing amount of evidence indicating that expression of genes related to inflammatory processes and the disbalance between the levels of pro- and anti-inflammatory molecules represent some of the fundamental mechanisms of aging. However, the actual role of inflammation in reproductive aging is poorly understood. It has been reported that low levels of serum androgens in elderly men correlate with increased expression of circulating pro-inflammatory cytokines IL1β, IL6 and TNFα [40,41]. These cytokines are also locally produced in testicular MACs during inflammation [23,42]. Although the testis is considered an immune-privileged organ it is quite evident that immune cells do gain access to this tissue. Furthermore, some of them also undergo local proliferation in the gonad. Under normal conditions, leukocytes, including T cells, natural killer cells, mast cells, eosinophils and MACs are mainly present within the testicular interstitium [43]. Among these, testicular MACs constitute the largest population.

MACs display a wide variety of phenotypes, depending on their tissue localization, the cytokine environment, and the time point in the inflammatory process. Consequently, MACs comprise multiple heterogeneous subsets [44]. CD163, CD68 and Iba1 are common tissue MAC biomarkers. Regarding CD163 expression, this cell-surface glycoprotein member of the scavenger receptor cysteine-rich superfamily identifies a subpopulation of resident MACs mainly associated to the maintenance of immune privilege [44]. On the other hand, monocytes recently arrived to the tissue from circulation express the lysosomal glycoprotein CD68 [44). CD68+ testicular MACs show a pro-inflammatory profile based on their ability to secrete pro-inflammatory cytokines [43]. Iba1 is a pan-MAC marker. All subpopulations of MACs examined to date, except alveolar MACs, express the Iba1 antigen [45].

In our experimental mouse models with altered longevity, we observed a negative association between longevity and the number of Iba1-immunopositive testicular MACs. In addition, CD68 and CD163 expression was increased in GH-Tg short-lived mice testes but reduced in GHRH-KO long-lived mice testes. Even though there was a trend toward lower CD68 and CD163 mRNA expression levels in the Ames dwarf testes, no actual statistically significant differences were observed.

In parallel with the influence of aging on testicular COX2 expression, we found an inverse association between longevity and PGD2 production in mouse testes. Previously, PGD2 has been implicated in the modulation of the migration capacity of murine MACs [46]. This raises the possibility that local production of PGD2 contributes to tissue recruitment of MACs. Therefore, the increased number of testicular MACs observed in mice with reduced longevity could, at least in part, be related to higher testicular levels of PGD2. Further analysis is required to address this matter. Overall our results indicate that a significantly increased inflammatory status of the testis, evidenced by a rise in the local MAC population, as well as by an increment in the expression of COX2 and the production of PGD2, is associated with decreased longevity.

Under physiological conditions, the immunosuppressive testicular microenvironment protects germinal cells from being attacked by the immune system. However, in inflammatory conditions, this tolerance is disrupted and immune cells and their mediators respond to germinal cell self-antigens, inducing damage to the germinal epithelium [44]. Thus, we set out to study testicular damage by examining peroxidative processes induced by oxidative stress, as it is currently regarded as one of the most important causes of impaired testicular function. The TBARS assay was used to estimate testicular lipid peroxidation and, indirectly, ROS production. In this context, it is important to bear in mind that TBARS also react with non-lipid molecules making the assay a non-specific marker of membrane lipid peroxidation [47]. TBARS production was significantly induced in GH-Tg short-lived mice testes compared to normal littermates, while decreased levels of lipid peroxidation were detected in GHRH-KO mice with extended longevity. These results are in agreement with other reports comprising different aging tissues including liver, kidney and brain in which decreased oxidative damage was seen in the long-lived Ames dwarf and GHRKO mice, and increased oxidative stress was observed in GH-Tg mice [48,49]. Regarding the testis, similar findings have been observed in aged Leydig cells where enhanced oxidative stress and peroxidative cell damage were reported [27,50].

Lipid peroxidation is a process mainly generated by the effect of several ROS. A major, although not the only, source of ROS production is energy transduction in the mitochondria [47]. Oxygen in mitochondria is reduced to water in four sequential steps that additionally generate ROS as intermediates. In fact, approximately 1% to 5% of the oxygen consumed by mitochondria is converted to ROS. A number of studies have demonstrated that mitochondrial integrity declines as a function of age, thus implicating mitochondria as a main source and target of free-radicals that culminate into the process of aging [47]. Increased oxidative tissue damage during aging has been explained by the existence of a reduced number of efficient mitochondria with electron transport tightly coupled to ATP production [51]. Also, several components of the oxidative phosphorylation system were reported to be elevated in long-lived Ames dwarf mice kidney and liver mitochondria predicting enhanced mitochondrial function and efficiency, two factors likely contributing to life span [52].

It is worth considering that ROS are produced in Leydig cells by the mitochondrial electron transport chain, as in other cells, but additionally, by the P450 enzymes involved in steroidogenesis [53]. Thus, the risk of oxidative damage from lipid peroxidation is especially high in the testis. However, basal levels of antioxidant defense systems in the male gonad seem to suffice to keep the natural occurring ROS at bay.

Mammalian cells are equipped with both enzymatic and non-enzymatic antioxidant defense mechanisms to cope with oxygen free radicals [54]. The testis is not an exception. In consequence, lipid peroxidation is modulated not only by the amount of ROS produced but also by the antioxidant system. Antioxidant enzymes such as superoxide dismutase 1 (SOD1), glutathione peroxidase (GPX), catalase (CAT) and peroxiredoxin 1 (PXR1) play major roles. In this context, cytosolic SOD1 is responsible for dismutation of superoxide anion (O_2<sup>-</.sup>_) to hydrogen peroxide (H_2_O_2_). It is generally accepted that GPX and PXR1 are the main routes for H_2_O_2_ catabolism when it is present at relatively low levels, whereas at higher H_2_O_2_ levels CAT would have a predominant role [54-56]. In the testis, GPX expression seems to be mainly restricted to germ cells [57] while CAT expression is primarily localized in peritubular and interstitial cells [58,59]. PXR1 expression has been described in peritubular, Leydig, Sertoli and spermatogenic cells [59,60].

In this study, we did not observe any changes in the testicular expression of SOD1, PXR1, CAT or GPX antioxidant enzymes in GHRH-KO and Ames dwarf mice with extended longevity. However, when short-lived (GH-Tg) mice testes were studied, we detected high levels of SOD1, PXR1 and CAT expression. These results, together with the increased TBARS production, suggest that in testes of adult mice with lower life expectancy, oxidative stress activates the enzymatic defense mechanisms to prevent further injury.

The fact that there are so many factors capable of inducing oxidative stress in the testes strongly suggests that this is a vulnerable tissue that, although it depends highly on oxygen to drive spermatogenesis, yet it is extremely susceptible to the toxic effects of reactive oxygen metabolites that accumulate in an age-related manner. It has been suggested that oxidative stress and apoptosis may be functionally linked. Apoptosis is a form of programmed cell death needed not only for elimination of those cells that represent a threat to the integrity of the organism but also for proper cellular development. In the testis, proliferation and apoptosis of germ cells occur during embryonic and postnatal periods and are sustained during the adulthood and constitute the basis of spermatogenesis [44]. Increasing amount of evidence suggests that ROS can induce apoptosis in germ cells [61]. Thus, given the clear effect of aging on testicular TBARS production, we decided to analyze whether variations in longevity could affect the occurrence of apoptotic events in the testis. When germ cells were examined by TUNEL assay, testes from mice with reduced longevity (GH-Tg) showed a marked increase in the number of apoptotic cells. In contrast, only a few apoptotic germ cells were detected in mice with increased longevity, suggesting that germ cell apoptosis is associated with aging. Our results are in agreement with recent data reporting an increased apoptotic index during aging in the human testis [15]. In addition, in extra-gonadal tissues including kidney and skeletal muscle, recent reports have described a decreased expression of apoptosis-related genes and pro-apoptotic proteins in long-lived GH receptor knockout mice [14].

In general, two major apoptotic pathways exist: the intrinsic or mitochondrial pathway and the extrinsic or death receptor pathway, triggered by internal and external signals, respectively [62]. Caspases belong to a cysteine-aspartic acid protease family whose sequential activation plays a central role in the execution-phase of cell apoptosis. Caspases exist as inactive pro-enzymes that undergo proteolytic processing at conserved residues to produce the active enzyme. Caspase-3 is an executioner caspase activated in both apoptotic cell pathways [62].

In this study, we have detected reduced levels of activated caspase-3 in GHRH-KO and Ames dwarf long-lived mice testes. In contrast, testes from short-lived (GH-Tg) mice show a significant increase in levels of both pro- and cleaved-caspase-3. Thus, taking into account the rise in the production of TBARS observed in testes of short-lived mice, our results strongly support a functional connection between oxidative stress and apoptosis in testes of mice with reduced longevity. In this context, up-regulation of caspase 3 activity was observed in rat germ cell cultures incubated in the presence of H_2_O_2_ [61].

In summary, our work provides new insights into the development of local inflammatory processes, the oxidative state and the occurrence of apoptotic events in the testis during aging and longevity. Previous reports highlighted that extended longevity reduced inflammatory activity, oxidative stress and apoptosis in several peripheral tissues [11-14]. In this study we have demonstrated that short-lived mice over-expressing GH exhibit higher levels of COX2/PGD2/TBARS and increased numbers of testicular MACs. A recent study reported that certain PGs induced ROS generation in the human testis [25]. Importantly, ROS are also produced by immune cells [47]. Thus, inflammatory events which include PGD2 production and a higher population number of testicular MACs expressing COX2 might contribute to the development of locally elevated levels of ROS in testes of mice with lower life expectancy. Subsequently, oxidative stress seems to activate the enzymatic defense mechanisms to prevent further injury to the germinal epithelium. However, the amount of peroxidative damage appears to be so extensive that the enzymatic antioxidant defense system cannot cope and apoptotic events are triggered specially in germ cells. In contrast, levels of COX/PGD2/TBARS and the numbers of testicular MACs and apoptotic germ cells are decreased in the remarkably long-lived Ames dwarf and GHRH-KO mice. In consequence, testes of adult mice with a higher life expectancy display anti-inflammatory, anti-oxidant and anti-apoptotic capacities.

Altogether, these studies on short- and long-lived mice have provided a greater understanding of the testicular physiology associated to the processes that drive aging.

## METHODS

### Animals

Three groups of mice were used in this study:

1. GH-Tg mice containing the bovine GH (bGH) gene fused to control sequences of the rat phospho-enolpyruvatecarboxykinase (PEPCK) gene. The hemizygous transgenic mice were derived from a founder male kindly provided by Dr T.E. Wagner and Dr J.S. Yun (Ohio University) and were produced by mating transgenic males with normal C57BL/6 × C3H F1 hybrid females purchased from the Jackson Laboratory (Bar Harbor, ME, USA) [29].
2. GHRH-KO mice produced on a mixed C57BL6 and 129SV background. Mice were derived from animals kindly provided by Dr. M. Alba and Dr. R. Salvatori (The Johns Hopkins University School of Medicine). The colony was generated by mating heterozygous males and females [30].
3. Ames dwarf (Prop1^df/df^) mice. In this colony, the Prop1 mutation is maintained on a heterogeneous genetic background by mating heterozygous females and homozygous mutant males [31].

Four to five mice per cage were housed under specific pathogen-free conditions in a room at 22±2°C with a controlled photoperiod of 12 h light:12 h dark cycle. Animals were given free access to water and nutritionally balanced diet (23.4% protein, 4.5% fat, 5.8% crude fiber; LabDiet, PMI Feeds, Inc., St. Louis, MO, USA). All experimental procedures were conducted with approval from the Southern Illinois University Institutional Animal Care and Use Committee following NIH guidelines. Adult mice were killed by cervical dislocation under isoflurane anesthesia according to protocols for Laboratory Animal Use. At the time of sacrifice, right testes were dissected and fixed for at least 48 h in formalin followed by dehydration, and then embedded in paraffin wax for histological and immunohistochemical studies, while left testes were preserved at −80°C for molecular biology analyses. Normal littermates from each group were used as controls.

### Tissue lysates and immunoblotting

Testes from adult mice were examined by immunoblotting. Testicular samples were lysed in RIPA buffer (150 mM NaCl, 50 mM Tris, 1 mM EDTA, 1% Nonidet P-40, 0.5% sodium deoxycholate, 0.1% sodium dodecyl sulfate, pH 7.4) supplemented with 1 mM sodium orthovanadate, 1 mM sodium fluoride and a commercial mixture of protease inhibitors (Roche Applied Science GmbH, Mannheim, Germany). Homogenates were kept in ice for 30 min, with occasional mechanical disruption using a pipet, followed by a centrifugation at 13,000 x g for 10 min. Supernatants were collected and assayed for protein content by the method described earlier [32]. Samples were used immediately or stored at −80°C until protein expression levels were determined by immunoblotting. Blots were performed as previously described [33] using 10 μg of protein lysates. Incubations were carried out using rabbit polyclonal anti-COX2 antiserum (1:250, Cayman Chemical #160106, Ann Arbor, MI, USA), rabbit monoclonal anti-catalase antibody (1:1000, Epitomics #2363-1, Burlingame, CA, USA), rabbit polyclonal anti-caspase 3 antiserum (1:250, Santa Cruz #sc-7148) or mouse monoclonal anti-actin antibody (1:5000, Calbiochem #CP01, La Jolla, CA, USA). Subsequently, the following peroxidase-labeled secondary antibodies were used: goat anti-mouse IgM serum (1:2000, Santa Cruz Biotechnology Inc. #sc-2064, Santa Cruz, CA, USA) for actin and goat anti-rabbit IgG serum (1:2500, Sigma-Aldrich #A0545, St Louis, MO, USA) for COX2, catalase, and caspase 3. Signals were detected with an enhanced chemiluminescence kit (BIORAD, Hercules, CA, USA).

### PGD2 assay

Testicular samples were lysed in supplemented RIPA buffer. PGD2 concentrations were determined using 100 μg protein aliquots and a commercially available kit as described elsewhere [33]. Proteins were precipitated by diluting the samples 1:1 with 0.4 N Perchloric Acid and incubating them in ice for 40 min. Following a centrifugation at 13,000 x g for 30 min, supernatants were collected and pre-treated with methoxylamine hydrochloride in order to prevent PG further chemical degradation.

The minimum detectable immunoassay concentration was 0.28 femtomole (fmol)/tube. Intra-assay and inter-assay coefficients of variation were less than 10% and less than 15%, respectively. Results were expressed as pmol PGD2/mg protein.

### Immunohistochemical analyses

Testes were fixed in 10% formalin, dehydrated and embedded in paraffin wax. Five μm sections obtained from three different levels were used for immunodetection of COX2, StAR and Iba1. In brief, testicular sections were deparaffinized and antigen retrieval by microwave irradiation in citrate buffer 0.01 M (pH 6.0) was performed for COX2 and Iba1 immunodetection. Endogenous peroxidase reactivity was quenched by a 20 min pre-treatment with 10% methanol, 0.3% H_2_O_2_ in *Phosphate Buffered Solution* PBS (for COX2 and StAR immunodetection) or 0.3% H_2_O_2_ in methanol (for Iba1 immunodetection). For StAR immunodetection, samples were permeabilized by a 5 min incubation with 0.5% saponin. Non-specific proteins were blocked by subsequent incubation for 30 min with a protein block buffer (5% goat normal serum prepared in PBS for immunodetection of COX2 and StAR or 5% BSA prepared in PBS for immunodetection of Iba1). After several wash steps, incubation with the antiserum (polyclonal rabbit anti-COX2 serum, 1:250, Cayman Chemical; polyclonal rabbit anti-StAR serum, 1:500, kindly provided by Dr. D. Stocco at Texas Tech University, Lubbock, TX, USA; or polyclonal rabbit anti-Iba1 serum, 1:1500, Wako Pure Chemical Industries Ltd. #019-19741, Osaka, Japan) diluted in incubation buffer (2% goat normal serum in PBS for immunodetection of COX2 and StAR, or 5% BSA, 0.1% Triton prepared in PBS for immunodetection of Iba1) was carried out in a humidified chamber at 4°C for 18h (for immunodetection of COX2 and StAR) or 3 days (for immunodetection of Iba1). Testicular sections were washed and incubated for 2h at room temperature with biotinylated secondary antiserum (goat anti-rabbit IgG serum, 1:200 for immunodetection of Iba1 and 1:500 for immunodetection of COX2 and StAR from Vector Laboratories Inc., Burlingame, CA, USA) diluted in incubation buffer (2% goat normal serum in PBS for immunodetection of COX2 and StAR or 5% BSA 0.1% Triton prepared in PBS for immunodetection of Iba1). Finally, immunoreactions were visualized with a 0.01% H_2_O_2_ and 0.05% 3,3-diaminobenzidine (DAB) solution (in 0.05 M Tris-HCl, pH 7.6) and an avidin-biotin-peroxidase system (Vector Laboratories Inc.).

For control purposes, either the first antiserum was omitted or incubation was carried out with normal non-immune sera.

Testicular quantification of Iba1-immunoreactive MACs was performed using a Zeiss microscope (Jena, Germany) with 400X magnification and a gridded eyepiece. In each testicular section, all fields were evaluated. The results were expressed as Iba1-immunoreactive cells/mm^2^ and Iba1-immunoreactive cells/tubule.

### Laser capture microdissection and RT-PCR analyses

Testicular sections from GH-Tg mice, GHRH-KO and Ames dwarf mice as well as their corresponding normal littermates were used. Sections were deparaffinized and immunostained with anti-COX2 antiserum (Cayman Chemical) as described above. Subsequently, laser capture microdissection (LCM) was performed as described earlier [34]. RNA from COX2-immunoreactive cells was extracted using the Paradise Plus Reagent system (Applied Biosystems, Foster City, CA, USA) following the manufacturer's instructions. Reverse transcription (RT)-reactions were performed using 500 ng total RNA and dN6 random primers as described previously [33]. RT-PCR analyses were performed using oligonucleotides for: CD68 (1° set: 5′-TGTCCTTCCCACAGGCAGCA and 5′-AGAGCAGG TCAAGGTGAACAG; nested-2° set: 5′-TGTCCTTCC CACAGGCAGCA and 5′-TGCATTTCCACAGCAGA AG) and StAR (5′-CCGGAGCAGCGTGGTGTCA and 5′-CAGTGGATGAAGCACCATGC). PCR conditions were 95°C for 5 min, followed by cycles of 94°C for 1 min, 55-60°C (annealing temperature) for 1 min and 72°C for 1 min, and a final incubation at 72°C for 5 min. PCR products were separated on 2% agarose gels, and visualized with ethidium bromide. The identity of the cDNA products was confirmed by sequence analysis on an ABI 373A DNA sequencer (Applied Biosystems).

### Real time-PCR analyses

Total RNA was prepared from testicular lysates using TRIzol Reagent (Invitrogen, Valencia, MO, USA) following the manufacturer's instructions. A pre-incubation of the extracts with RNase-free DNase (1 unit per μg RNA, Promega Corporation, Madison, WI, USA) at room temperature for 20 min ensured degradation of contaminating genomic DNA. RT-reaction was performed using 500 ng total RNA and dN6 random primers.

Real time-PCR assays were performed as described elsewhere [33] using oligonucleotide primers for CD68 (5′-TGTCCTTCCCACAGGCAGCA and 5′-TGCATTT CCACAGCAGAAG), CD163 (5′-AGCTGGGATGCC CAACT and 5′-CAAAGAGCTGACTCATTC), StAR (5′-CCGGAGCAGCGTGGTGTCA and 5′-CAGTGGA TGAAGCACCATGC), superoxide dismutase 1 (5′-AAAGCGGTGCGTGCTGAA and 5′-CAGGTCTCCA ACATGCCTCT), catalase (5′-CCGACCAGGGCATC AAAA and 5′CATTGGCGATGGCATTGA), peroxire-doxin 1 (5′-CACCCAAGAAACAAGGACCA and 5′-GAGATACCTTCATCAGCCTT), glutathione peroxi- dase (5′-CCTCAACTACGTCCGACCTG and 5′-CAA TGTCGTTGCGGCACACC) and GAPDH (5′-GACGG CCGCATCTTCTTGT and 5′-ACCGACCTTCACCAT TTTGTCT). Reactions were conducted using SYBR Green PCR Master Mix and the ABI PRISM 7500 sequence detector System (Applied Biosystems). The reaction conditions were as follows: 10 min at 95°C (one cycle), followed by 40 cycles of 20 s at 95°C, 30 s at 55°C and 1 min at 72°C for CD68, superoxide dismutase 1 and glutathione peroxidase; 10 min at 95°C (one cycle), followed by 40 cycles of 20 s at 95°C and 1 min at 55°C for CD163; 10 min at 95°C (one cycle), followed by 40 cycles of 20 s at 95°C, 30 s at 55°C and 1 min at 60°C for StAR, catalase and pero-xiredoxin 1 and 10 min at 95°C (one cycle), followed by 40 cycles of 20 s at 95°C and 1 min at 60°C for GAPDH.

Following a mathematical model [35], the relative levels of mRNA expression were determined for each sample using GAPDH as the housekeeping gene.

### Thiobarbituric Acid Reactive Substances (TBARS) assay

Lipid peroxidation was assessed by determining the production of Thiobarbituric Acid (TBA)-Reactive Substances, which mainly detects malondialdehyde (MDA). Approximately 3-4 mg of testicular tissue were resuspended in 100 μl of 0.4% butylated hydroxytoluene prepared in (PBS) and then disrupted by ultrasonic irradiation. TBARS determination was performed using 25 μl of total cell extract and 175 μl of a pre-formed reaction mix containing 0.15% SDS, 0.5 N HCl, 0.75% phosphotungstic acid and 0.175% TBA. Samples were boiled for 45 min followed by a 5 min centrifugation at 4°C (14,000 x g). Supernatants were extracted with 200 μl of n-butanol. MDA-TBA complexes present in the organic phase were measured colorimetrically at 532 nm. Standard curve (0.48-15.68 μM) was built using MDA generated from 1,1,3,3-tetramethoxypropane. Results were expressed as pmol MDA/mg protein.

### TUNEL assay

Cleavage of genomic DNA during apoptosis was assessed using In Situ Cell Death Detection Kit, POD (Roche Applied Science GmbH). Testicular sections were deparaffinized and hydrated by descending series of isopropanol. Sections were then permeabilized with 20 μg/ml Proteinase K in 10 mM Tris–HCl, pH 7.4 for 15 min at room temperature, followed by incubation with blocking solution (3% H_2_O_2_ in methanol) for 10 min at room temperature. After being rinsed twice, samples were incubated for 1h at 37°C (in a humidified chamber) with TUNEL reaction mix. Reaction mix contains the labeling solution (fluorescein-nucleotide mixture) and the enzyme solution (terminal deoxynucleotidyl transferase -TdT-). Sections were then rinsed three times followed by incubation for 30 min at 37°C (in a humidified chamber) with a horse radish peroxidase-conjugated anti-fluorescein antibody. The signal was finally detected by adding a 0.01% H_2_O_2_ and 0.05% 3,3-diaminobenzidine (DAB) solution (in 0.05 M Tris-HCl, pH 7.6). For control purposes, the TdT enzyme solution was omitted in the TUNEL reaction mix.

### Statistical analyses

Statistical analyses were performed using Student's t test for comparisons of two means.

Data are expressed as mean + S.E.M.

For immunoblotting studies, bands were quantified by densitometry and normalized to actin using ImageJ (ImageJ, U.S. National Institutes of Health, Bethesda, MA, USA, http://imagej.nih.gov/ij/).
